# Optimizing support vector machines and autoregressive integrated moving average methods for heart rate variability data correction

**DOI:** 10.1016/j.mex.2023.102381

**Published:** 2023-09-16

**Authors:** Jakob Svane, Tomasz Wiktorski, Stein Ørn, Trygve Christian Eftestøl

**Affiliations:** a Department of Electrical Engineering and Computer Science, University of Stavanger, Stavanger 4021, Norway; b Division of Cardiology, Stavanger University Hospital, Stavanger 4011, Norway

**Keywords:** HRV, Arima, SVR, Hyperparameters, Artifacts, Correction, Optimization of ARIMA and SVR for HRV Data Correction

## Abstract

Heart rate variability (HRV) is the variation in time between successive heartbeats and can be used as an indirect measure of autonomic nervous system (ANS) activity. During physical exercise, movement of the measuring device can cause artifacts in the HRV data, severely affecting the analysis of the HRV data. Current methods used for data artifact correction perform insufficiently when HRV is measured during exercise. In this paper we propose the use of autoregressive integrated moving average (ARIMA) and support vector regression (SVR) for HRV data artifact correction. Since both methods are only trained on previous data points, they can be applied not only for correction (i.e., gap filling), but also prediction (i.e., forecasting future values). Our paper describes:

• why HRV is difficult to predict and why ARIMA and SVR might be valuable options.
• finding the best hyperparameters for using ARIMA and SVR to correct HRV data, including which criterion to use for choosing the best model.
• which correction method should be used given the data at hand.

## Graphical abstract

**Figure:**
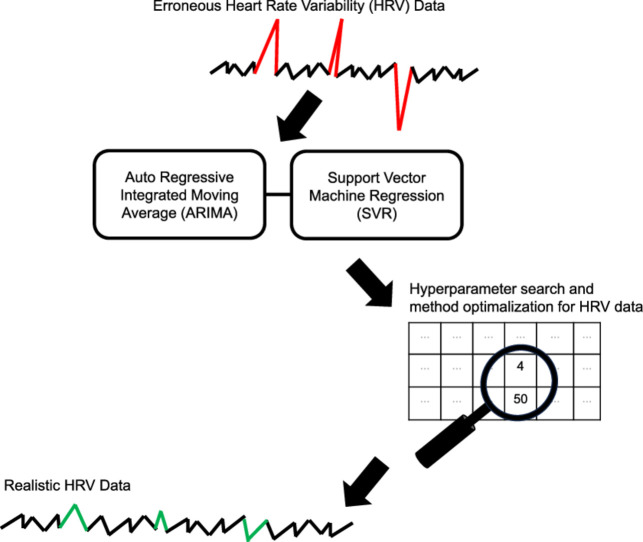

Specifications table

**Table:** 

Subject area:	Bioinformatics
More specific subject area:	Heart Rate Variability Data Analysis
Name of your method:	Optimization of ARIMA and SVR for HRV Data Correction
Name and reference of original method:	O'Donovan, T., Short term forecasting: an introduction to the Box-Jenkins approach, John Wiley & Sons Ltd, 1983. Vapnik, V., The nature of statistical learning theory, Springer, 1999.
Resource availability:	Vollmer, M., Bläsing, D., Reiser, J. E., Nisser, M., & Buder, A. (2022). Simultaneous physiological measurements with five devices at different cognitive and physical loads (version 1.0.1). PhysioNet. https://doi.org/10.13026/zhns-t386.

## Method details

### Rationale

Heart rate (HR) variability (HRV) has recently gained considerable attention in both medical and sporting contexts, as it functions as an accessible, non-invasive proxy for both autonomic nervous system (ANS) function and heart function [[1], [2]]. HRV is the variation in time difference between heartbeats, with the time difference between two heartbeats referred to as the RR interval length. HRV is often measured during rest with the patient in fixed position to ensure reliable and standardized measurements as well as constant HR, circumventing the dependency of HRV on HR [3]. However, recently Búzás et al. [4] developed a method which might aid significantly in the analysis of HRV data measured during physical activity, during which HR increases and varies significantly compared to during rest.

When measured during movement, either by photoplethysmography (PPG) or with a heart rate chest strap, motion artifacts present a significant problem for the analysis of the HRV data. These artifacts need to be handled correctly, as they can greatly affect the analysis [5], [6]. Bourdillon et al. [7] showed that an isolated artifact on a 4-min window can alter HRV metrics by as much as 413%. Typically, artifacts are detected by use of median filters, and then corrected using various gap filling methods. The most common method for gap filling is cubic spline interpolation [8]. Other methods have also been proposed [5], [9], including linear interpolation and simple deletion, but the error for longer gaps is usually large and the beat-to-beat variability is in many cases unrealistic for the corrected HRV. We have previously tested recurrent neural networks (RNNs) for HRV data artifact correction [10], but did not achieve satisfactory results. In the article, transfer learning (TL) was suggested as a remedy for improving results and reducing training data. We have since tested TL, but the results did not improve notably, and are therefore not reported. Thus, although filters perform adequately for artifact detection, there is no consensus on how to properly replace the erroneous values. Furthermore, methods for artifact correction may also be used for forecasting if they only use previous data points for prediction (as opposed to using both previous and future points). Forecasting RR interval length (or heart rate) can possibly aid in diagnosis and treatment decisions preceding/following acute heart dysfunction [11]. In this paper the term correction will be used to encompass both prediction and correction.

### Approach

Box-Jenkins methods, specifically autoregressive integrated moving average (ARIMA), is one of the most used timeseries forecasting methods [12]. Unlike most machine learning methods, ARIMA is a simple method that does not require large amounts of training data. The performance and simplicity of the method establishes a strong argument for assessing it as a HRV data artifact correction method. Still, due to the dynamics and interplay of the heart and the ANS, HRV data is highly volatile, so that other more sophisticated methods should also be considered. As mentioned, neural networks for timeseries forecasting were assessed in [10]. Several studies have shown that support vector machine regression (SVR) can outperform other methods for volatile time series forecasting [[13], [14], [15]]. For both ARIMA and SVR, several hyperparameters must be determined before training the model. These are parameters that control how the methods work, and greatly affect the results. This paper aims to find the hyperparameters that yield the best results when using ARIMA and SVR for HRV data correction.

When analyzing HRV data, it is common to summarize the time series with both time domain metrics and frequency domain metrics. In this paper, we will consider the root mean square of successive differences (RMSSD) and the standard deviation of all normal-normal intervals (SDNN) in the time domain. Normal-normal intervals are simply error-free RR intervals. We will also consider three frequency bands: very low frequency (VLF), 0Hz-0.04 Hz; low frequency (LF), 0.04Hz-0.15 Hz, high frequency (HF), 0.15Hz-0.4 Hz; and total power (TP), the sum of the power of these three bands. For this, the RR intervals are resampled evenly by applying interpolation, and the frequency domain metrics are calculated using the Fast Fourier Transform (FFT).

The data sets used in this paper are from two different databases. From the North Sea Race Endurance Exercise Study (NEEDED) we have HRV data from 17 different participants during various stages of a bicycle race [16]. From the Physionet simultaneous physiological measurements, we have HRV data from 12 different individuals during a 5-minute period of incline treadmill walking [17], [18]. The NEEDED data contains both healthy subjects and subjects with varying degrees of atherosclerosis, whereas the Physionet data set consists of only healthy subjects. These are the same data sets used in Svane et al. [10], in which the preprocessing of the data is thoroughly detailed.

## Methods

### ARIMA

ARIMA is a forecasting method used for prediction of future outcomes based on previous observations in a time series. It is one of the most widely used approaches for time series forecasting [12]. To build an ARIMA model, we combine an autoregressive model with a moving average model and include differencing as part of the model.

First, to remove non-stationarity from the time series, we can apply differencing. For a value $x_{t}$, where the subscript *t* denotes the *t*’th observation, we have

(1) $$x_{t}^{\left(d\right)}=x_{t}^{\left(d-1\right)}-x_{t-1}^{\left(d-1\right)}.\;$$

The superscript *d* denotes the order of differencing.

An autoregressive model is a model that predicts a variable based on a linear combination of past values of the same variable. A model of order *p* can be written as

(2) $$x_{t}=c+\;\phi_{1}x_{t-1}+\phi_{2}x_{t-2}+\ldots +\phi_{p}x_{t-p}+\varepsilon_{t},\;$$

where $\phi_{i}$ is the weight given to the *i*’th back lagged value, c is a constant term and $\varepsilon_{t}$ is the error.

A moving average model uses the past forecasting errors to predict the current variable. A model of order *q* can be written as

(3) $$x_{t}=c+\;\varepsilon_{t}+\theta_{1}\varepsilon_{t-1}+\theta_{2}\varepsilon_{t-2}+\ldots +\theta_{q}\varepsilon_{t-q},\;$$

where $\theta_{i}$ is the weight given to the error of the *i*’th prediction, c is a constant term and $\varepsilon_{i}$ is the error for the *i*’th prediction.

By combining these three parts, we get

(4) $$x_{t}^{\left(d\right)}=c+\;\phi_{1}x_{t-1}^{\left(d\right)}+\phi_{2}x_{t-2}^{\left(d\right)}+\ldots +\phi_{p}x_{t-p}^{\left(d\right)}+\theta_{1}\varepsilon_{t-1}+\theta_{2}\varepsilon_{t-2}+\ldots +\theta_{q}\varepsilon_{t-q}+\varepsilon_{t}.\;$$

The ARIMA model is trained and fitted on a given number of previous data points, which we will call sequence length. Thus, the four hyperparameters for an ARIMA model are *p* - order of the autoregressive part; *d* - degree of differencing; *q* - order of the moving average part; and sequence length – the number of points the model is trained on. A detailed explanation of ARIMA can be found in [19].

### SVR

Support vector machines (SVM) are supervised learning models, first developed by Vladimir Vapnik [20]. Most commonly, they are used for classification tasks, but they have also been applied successfully for regression tasks [[13], [14], [15]]. When used for regression, the method is usually referred to as support vector regression. By non-linearly mapping the input data into a high-dimensional feature space, then performing a linear regression in this space, a non-linear regression line in the input space can be found.

Consider a set of data points $\left(x_{1},y_{1}\right),\;\left(x_{2},y_{2}\right)$, …, $(x_{m},y_{m}),\;$where $x_{i}\in R^{n}\;$are training inputs, $y_{i}\in R\;$are training outputs, and the subscript m is the total number of samples. SVR approximates the output with the function

(5) $$y\left(x\right)=w^{T}\Phi \left(x_{i}\right)+b,$$

where $w^{T}$ is the transposed weight vector, $\Phi (x_{i})$ is the high dimensional feature space mapped from the input space $x_{i}$, and b is the bias. The goal is to find a function y(x) that fits the targets $y_{i}$, with a maximum acceptable deviation ε, also referred to as the margin. By introducing slack variables $\xi_{i}^{+}$ and $\xi_{i}^{-}$, we allow data points to fall outside this margin. These variables denote the distance from the given data point to the margin, with the + and – subscripts denoting data points above and below the margin, respectively. By multiplying these variables with a user defined regularization constant C, we can decide the importance of the points lying outside the margin. The optimization problem becomes

(6) $$\begin{array}{c} \text{minimize}\;\frac{1}{2}w^{T}w+C\sum_{i=1}^{m}\left(\xi_{i}^{+}+\xi_{i}^{+}\right),\;\;\\ \text{subject}\;\text{to}\;\left\{ \begin{array}{c} y_{i}-\left(w^{T}\Phi \left(x_{i}\right)+b\right)\leq \varepsilon +\xi_{i}^{+},\\ \left(w^{T}\Phi \left(x_{i}\right)+b\right)-y_{i}\leq \varepsilon +\xi_{i}^{-},\\ \xi_{i}^{+},\xi_{i}^{-}\geq 0. \end{array}\right. \end{array}$$

Increasing C will decrease bias but increase variance, whereas decreasing C will increase bias but decrease variance. To solve Eq. (6), Lagrangian multipliers are introduced, finally yielding

(7) $$y\left(x\right)=\;\sum_{i=1}^{m}\left(a_{i}^{+}-a_{i}^{-}\right)K\left(x,x_{i}\right)+b,\;$$

where $a_{i}^{+}$ and $a_{i}^{-}$ are Lagrangian multipliers and the kernel function is defined as

$K\left(x,x_{i},\right)=\;\Phi \left(x\right)\cdot \Phi \left(x_{i}\right)$. The kernel function maps the input space into a high-dimensional feature space, making it possible to find a non-linear regression line in the input space, without the need of explicitly computing the mapping Φ(x). Any function can be used as the kernel function if it satisfies Mercer's condition [20]. The kernels explored in this paper are the linear, polynomial, sigmoidal and radial basis function (RBF) kernels, as detailed below.

(8) $$\begin{array}{c} \text{Linear}\;\;:\;K\left(x,x_{i}\right)=x\cdot x_{i},\\ \text{Polynomial}\;:\;K\left(x,x_{i}\right)={\left(\gamma x\cdot x_{i}+r\right)}^{\beta },\\ \text{Sigmoidal}\;:\;K\left(x,x_{i}\right)=\tanh\left(\gamma x\cdot x_{i}+r\right),\\ \text{RBF}\;:\;K\left(x,x_{i}\right)=e^{-\gamma {∥x-x_{i}∥}^{2}}. \end{array}$$

Here γ, r and β are user defined hyperparameters that need to be determined before training the model. In addition to these three parameters, the number of back-lagged observations used to predict the next data point must be determined. We will call this the sequence length. Lastly, by differencing the data before training the model, we can remove non-stationarity and possibly improve the method. The order of differencing is denoted by the parameter *d,* as for ARIMA*.*

For predicting several points in sequence using SVR, we will apply a chained multioutput regression (CMR). CMR works by predicting the first point, then using this prediction as input for the next prediction, and so on until you have predicted the desired number of points. A detailed explanation of SVR can be found in [21].

### Hyperparameter search

To find the best hyperparameters for both SVR and ARIMA, a hyperparameter search was carried out. Different combinations of parameters were assessed in a grid search, with the range of values tested for SVR and ARIMA presented in Tables 1 and 2, respectively. Both methods were tested on HRV data from 16 participants from the NEEDED study and 11 participants from the Physionet data. For each data set, the SVR models were trained on a range of number of samples to see how the training set size affected the results, whereas the ARIMA models were trained on data of length equal to the sequence length. Both methods were tested on the same test sets of 250 samples. The number of test samples were chosen to obtain test sets of approximately one to two minutes, which is a common time frame for ultra-short-term HRV recordings [22], [23]. For each test set, a gap of length 10 was introduced in the middle of the test set, which was then filled by the SVR prediction, ARIMA prediction, cubic spline interpolation, linear interpolation, and simple deletion. The most common HRV metrics were calculated for each method of gap filling, and the relative error was reported.

**Table 1:** Scope of hyperparameter search for ARIMA models.

Parameter	Values						
*p*	1	2	3	4	5		
*d*	0	1	2				
*q*	1	2	3	4	5		
Sequence length	5	10	15	20	30	50	100

**Table 2:** Scope of hyperparameter search for SVR models.

Parameter	Values						
*C*	0.1	1	10	100			
ε	0.01	0.05	0.1	1			
K(·)	Linear	Polynomial	Sigmoidal	RBF			
γ	0.1	1	10	100			
β	3	4	5				
*r*	0	1					
*d*	0	1	2				
Sequence length	5	10	15	20	30	50	100

To test whether there is any non-random structure left in the signal after differencing, we also explored simply adding white noise to the differenced signal as a method for gap filling. Búzás et al. [4] found that, for a given HR, the difference between successive RR intervals showed a quasi-Gaussian distribution. Assuming a quasi-Gaussian distribution for the NEEDED and Physionet data as well, we added Gaussian noise based on the standard deviation of the differenced data, with the results of this method reported together with the other methods.

Choosing the hyperparameters that give the best performing model, depends on how you define the performance. For the models in this paper, as for the models in [10], it is apparent that choosing the model with the smallest root mean square error of the predicted values seem to generally give good results. However, we are mostly looking to minimize the error in the predicted HRV metrics, not necessarily replicate the actual values of the RR intervals. In both [6] and [10], it is evident that the frequency domain metrics are harder to predict accurately than the time domain metrics, in particular the low and high frequency bands. This is also seen in the following results. Therefore, we have chosen the model that minimizes the error of LF and HF. Formally, we can write

(9) $$\Theta =\arg\mathrm{mi}n_{\Theta }\left(LF_{err}+HF_{err}\right).$$

Here, ${LF}_{err}\;\text{and}\;{HF}_{err}$ are the errors in LF and HF as a function of the hyperparameters Θ.

## Method validation

The hyperparameters yielding the best three models for ARIMA and SVR according to our criterion in Eq. (9), are displayed in Tables 3 and 4, respectively. Interestingly, for SVR, the linear kernel gives the best results. The sequence length for the best models is five, meaning that the predictions are based on only the previous five points. When using CMR to make several predictions in sequence, the linear kernel can still yield a non-linear relationship between the 10 predicted points, maintaining the variability of the data.

**Table 3:** Hyperparameters for the three best performing ARIMA models.

	*p*	*d*	*q*	Sequence length
ARIMA 1	2	2	4	30
ARIMA 2	3	2	4	15
ARIMA 3	4	2	4	15

ARIMA – Autoregressive Integrated Moving Average; p – order of autoregressive part; d – order of difference; q – order of moving average part; sequence length – number of data points used for training.

**Table 4:** Hyperparameters for the three best performing SVR models.

	*C*	ε	K(·)	γ	β	*r*	*d*	Sequence length
SVR 1	1	0.01	Linear	N.A	N.A	N.A	0	5
SVR 2	100	0.01	Linear	N.A	N.A	N.A	0	5
SVR 3	10	0.01	Linear	N.A	N.A	N.A	0	5

SVR – Support Vector Regression; N.A – not applicable; d – order of differencing; C,ε, K,γ, β, r – hyperparameters, see Eqs. (2-4) for interpretation.

**Fig. 1:**
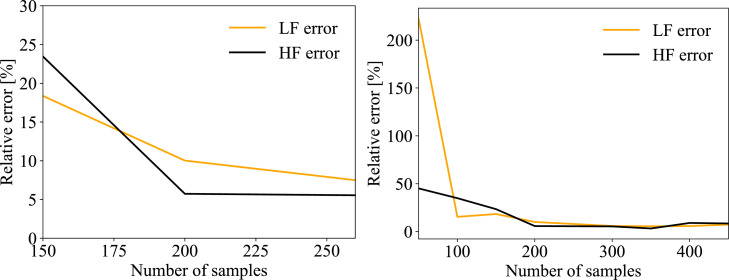
Left: Mean LF and HF relative error of all NEEDED participants as a function of training samples for SVR model. Right: Zoomed in around 200 samples.

**Fig. 2:**
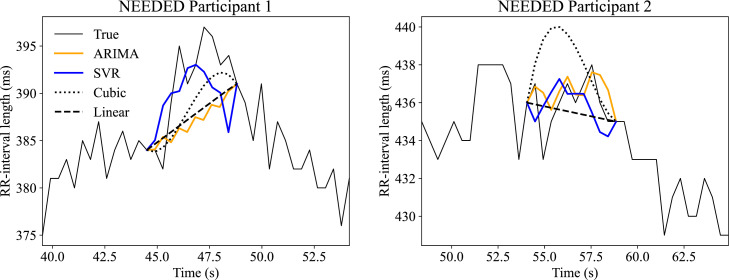
RR interval length predictions for ARIMA, SVR, cubic interpolation and linear interpolation for two different NEEDED participants. ARIMA = autoregressive integrated moving average; SVR = support vector regression.

The SVR models were trained on a range of numbers of training samples to determine how much training data is strictly necessary. Fig. 1 shows how the relative error of LF and HF changes based on the number of training samples. For LF error, there is a large drop in from 50 to 100 samples, and a gradual decrease from 100 to 200 samples. The error continues to decrease for more samples, although very slowly. For HF error, there is a steady decrease until around 200 samples, at which point the error levels out. Increasing the number of samples above 200 might give better results, but would also necessitate a longer segment of error free data to train the model. Thus, it seems the best tradeoff between error and number of samples is around 200. Therefore, the results in the following section are based on models trained on 200 samples. Figs. 1 and 2 are based on the best SVR model and might differ for other models.

The relative error for RMSSD, SDNN, VLF, LF, HF and TP from the best model with ARIMA and SVR, in addition to simple deletion, linear and cubic interpolation, and Gaussian noise on the NEEDED and Physionet data are reported in Tables 5 and 6, respectively. Clearly, the errors for the time domain metrics are very low for each method, whereas the frequency domain metrics vary to a larger extent, as referred to in Eq. (5). We see that ARIMA, SVR and linear interpolation perform relatively equally on both the NEEDED and the Physionet data, while cubic interpolation and deletion performs a lot worse. The method of adding Gaussian noise performs the worst on nearly every metric. There does not seem to be any differences in the results for data from healthy subjects and data from subjects with atherosclerosis.

**Table 5:** Mean relative error in HRV metrics with different methods on NEEDED data.

	RMSSD	SDNN	VLF	LF	HF	TP
ARIMA	1.2%	0.81%	2.9%	5.8%	7.0%	3.1%
SVR	1.1%	0.54%	2.2%	10%	5.7%	0.18%
Deletion	1.0%	1.8%	19%	49%	20%	15%
Linear interpolation	1.7%	0.69%	2.9%	4.3%	10%	2.4%
Cubic interpolation	1.3%	1.1%	5.8%	42%	84%	5.8%
Gaussian noise	2.5%	1.9%	10%	105%	176%	14%

ARIMA = Autoregressive Integrated Moving Average; SVR = Support Vector Regression; RMSSD = root mean square of successive differences; SDNN = standard deviation of NN intervals; VLF = very low frequency; LF = low frequency; HF = high frequency; TP = total power.

**Table 6:** Mean relative error in HRV metrics with different methods on Physionet data.

	RMSSD	SDNN	VLF	LF	HF	TP
ARIMA	1.3%	0.55%	2.3%	4.5%	8.1%	2.6%
SVR	1.3%	0.38%	1.8%	3.3%	7.1%	1.6%
Deletion	1.1%	1.8%	2.0%	46%	25%	15%
Linear interpolation	1.8%	0.72%	3.2%	4.1%	13%	2.6%
Cubic interpolation	1.3%	1.1%	7.0%	52%	108%	7.2%
Gaussian noise	2.0%	2.9%	25%	28%	59%	22%

ARIMA = Autoregressive Integrated Moving Average; SVR = Support Vector Regression; RMSSD = root mean square of successive differences; SDNN = standard deviation of NN intervals; VLF = very low frequency; LF = low frequency; HF = high frequency; TP = total power.

By inspection, it is evident that ARIMA, SVR and linear interpolation all perform well on almost every data set but perform dissatisfactory on one or two data sets. Table 7 shows each method's worst performance on an individual data set (not necessarily the same data set for each method) from the NEEDED data. Here, none of the methods perform acceptably, but ARIMA, SVR and linear interpolation still substantially outperform cubic interpolation and deletion. Considering the results in Table 7, the median errors are reported in Tables 8 and 9. We see that ARIMA, SVR and linear interpolation perform very well. Fig. 2 displays the predicted RR interval values on two participants from NEEDED. Although the RR interval predictions are not perfect for ARIMA and SVR, they seem to, visually, better preserve the general shape and variability of the RR interval curve than interpolation does. Neither linear nor cubic interpolation are able to recreate the variability when predicting several points in sequence. Evidently, visual reconstruction is not necessary for accurate representations of the HRV metrics we have reported in this paper, as Table 5, Table 6, Table 7, Table 8, Table 9 show both deletion and linear interpolation perform reasonably well despite a lack of visual similarity. However, it could be important for the calculation of other metrics [24], or for RR interval analysis using other methods (e.g., deep learning).

**Table 7:** The worst-case error in HRV metrics with different methods on data from NEEDED.

	RMSSD	SDNN	VLF	LF	HF	TP
ARIMA	2.8%	1.9%	7.3%	5.4%	52%	7.5%
SVR	0.56%	0.28%	0.39%	98%	8.4%	0.15%
Deletion	0.99%	1.0%	29%	215%	9.3%	29%
Linear interpolation	3.0%	1.7%	6.3%	5.6%	51%	6.7%
Cubic interpolation	2.7%	0.94%	11%	67%	620%	22%
Gaussian noise	12%	18%	93%	619%	1043%	185%

The results are not necessarily on the same data set. ARIMA = Autoregressive Integrated Moving Average; SVR = Support Vector Regression; RMSSD = root mean square of successive differences; SDNN = standard deviation of NN intervals; VLF = very low frequency; LF = low frequency; HF = high frequency; TP = total power.

**Table 8:** Median relative error in HRV metrics with different methods on NEEDED data.

	RMSSD	SDNN	VLF	LF	HF	TP
ARIMA	1.1%	0.41%	1.3%	5.2%	2.4%	1.2%
SVR	1.0%	0.29%	1.6%	4.0%	3.5%	1.4%
Deletion	1.0%	1.5%	13%	36%	12%	6.2%
Linear interpolation	1.4%	0.28%	0.10%	4.3%	4.8%	1.1%
Cubic interpolation	1.1%	0.87%	2.4%	9.8%	19%	1.7%
Gaussian noise	1.6%	0.42%	4.6%	14%	39%	4.8%

ARIMA = Autoregressive Integrated Moving Average; SVR = Support Vector Regression; RMSSD = root mean square of successive differences; SDNN = standard deviation of NN intervals; VLF = very low frequency; LF = low frequency; HF = high frequency; TP = total power.

**Table 9:** Median relative error in HRV metrics with different methods on Physionet data.

	RMSSD	SDNN	VLF	LF	HF	TP
ARIMA	1.2%	0.28%	1.0%	3.9%	1.5%	1.6%
SVR	1.1%	0.28%	1.6%	3.1%	3.8%	1.5%
Deletion	1.0%	1.6%	9.5%	44%	21%	6.1%
Linear interpolation	1.6%	0.29%	1.5%	4.0%	4.9%	1.5%
Cubic interpolation	1.2%	0.90%	2.4%	7.9%	26%	2.3%
Gaussian noise	1.1%	1.2%	8.8%	7.0%	49%	8.3%

ARIMA = Autoregressive Integrated Moving Average; SVR = Support Vector Regression; RMSSD = root mean square of successive differences; SDNN = standard deviation of NN intervals; VLF = very low frequency; LF = low frequency; HF = high frequency; TP = total power.

## Conclusion

The objective of this paper was to test and optimize ARIMA and SVR as artifact correction methods for HRV data during physical exercise. A hyperparameter search was carried out to find the optimal hyperparameters, and the methods were compared to the most used methods in the literature. Differencing was applied to the data sets, and it was found that minimizing LF and HF error seem to yield the best overall results for minimizing the total error of all HRV metrics. For the best ARIMA models, the sequence length was in the range 15–30, the order of differencing was zero, and the order of the moving average was two. The order of the autoregressive part was less influential. For the SVR models, the linear kernel performed the best, with a sequence length of five, zero order differencing and ε=0.01. The value of *C* appeared unimportant. Regarding training data for the SVR models, 200 samples seemed to give the best results for the fewest number of samples.

The mean relative error of the HRV metrics across all subjects was significantly better using ARIMA, SVR and linear interpolation than deletion, cubic interpolation, and Gaussian noise. For ARIMA, SVR and linear interpolation, the median error was even lower than the mean error, implying that these methods perform very well on most data sets. One poor data set might alter the mean error, as indicated in Table 7. Although linear interpolation performs reasonably well regarding the HRV metrics, the actual RR interval lengths predicted are unrealistic. Here, ARIMA and SVR outperform linear interpolation, with SVR possibly yielding the best results. The poor results from using Gaussian noise as a gap filling approach indicate that, even after detrending, there might be a non-random structure left in the HRV data that underpins the usefulness of machine learning methods for artifact correction.

When choosing the optimal way of correcting HRV data errors, various considerations should be made. With less than 200 error-free samples available, ARIMA might be a better choice than SVR, due to the training data requirements. If only the HRV metrics are of interest (i.e., the RR interval lengths are not important), linear interpolation can be an easy and acceptable approach. The same applies if time is very limited, for example if the training and the corrections need to be carried out in real time on a wearable device. With more data, SVR is probably the best choice, especially if the RR interval lengths are of interest. Interestingly, cubic interpolation – the most used method in the literature– appears to perform the worst. In some cases, if RMSSD is the only analyzed HRV metric, cubic interpolation can perform sufficiently. However, even in these cases, deletion is probably a better choice, since it performs better than cubic interpolation for RMSSD and does not alter the RR interval lengths to the same degree. Deletion is also the recommendation given in [5] and [6] for these instances. For the methods in this study, there does not seem to be a difference in the results when applied to data from healthy subjects or data from subjects with atherosclerosis.

## CRediT authorship contribution statement

**Jakob Svane:** Conceptualization, Methodology, Software, Formal analysis, Writing – original draft. **Tomasz Wiktorski:** Conceptualization, Methodology, Supervision, Writing – review & editing. **Stein Ørn:** Resources, Supervision, Funding acquisition. **Trygve Christian Eftestøl:** Supervision, Writing – review & editing.

## Declaration of Competing Interest

The authors declare that they have no known competing financial interests or personal relationships that could have appeared to influence the work reported in this paper.

## Data Availability

Some data is available, some in confidential. This is clearly stated in the article.
